# Evaluation of photobiomodulation in salivary production of patients with xerostomy induced by anti-hypertensive drugs: Study protocol clinical trial (SPIRIT compliant)

**DOI:** 10.1097/MD.0000000000019583

**Published:** 2020-04-17

**Authors:** Maria Lucia Zarvos Varellis, Marcela Leticia Leal Gonçalves, Vanessa Christina Santos Pavesi, Anna Carolina Ratto Tempestini Horliana, Daniela de Fátima Teixeira da Silva, Lara Jansiski Motta, Valdomiro F. Barbosa Filho, Cícero Dayves Silva Bezerra, Felipe Gonçalves da Silva, Sandra Kalil Bussadori, Alessandro M. Deana

**Affiliations:** Postgraduate Program in Biophotonics Applied to Health Sciences, University Nove de Julho (UNINOVE), Rua Vergueiro, Liberdade, São Paulo, Brazil.

**Keywords:** anti-hypertensive drugs, hypertension, photobiomodulation, saliva, salivary glands

## Abstract

**Introduction:**

Hypertension (systemic arterial hypertension [SAH]) is a systemic condition that affects about 30% of the world population, according to data from the World Health Organization (WHO). Drugs used to control this disease have the potential to induce xerostomia, an oral condition in which the decrease of the salivary flow is observed and whose presence leads to the increase of the index of caries, periodontal disease, loss of the teeth, dysgeusia, difficulty of mastication, dysphagia, bad breath and oral burning and impairment of prothesis installed in the buccal cavity, including retention of removable and total dentures.

**Methods:**

This is a randomized, placebo-controlled, blind clinical protocol that aims to analyze the impact of phobiomodulation (PBM) on salivary glands of patients with antihypertensive drug induced xerostomia. Patients will be divided into 2 groups: G1: older adults with xerostomia induced by antihypertensive drugs and treatment with PBM (n = 30); G2: placebo PBM (n = 30). The irradiation will be made using a diode laser emitting at 808 nm with 100 mW and 40 seconds of exposure per site at the salivary glands. Twenty sites will be irradiated weekly for 4 weeks. Non-stimulated and stimulated salivary flow will be analyzed before and after the treatment.

**Results:**

This protocol will determine the effectiveness of photodynamic therapy regarding the reduction of xerostomia in older adults using antihypertensive drugs.

**Conclusion:**

This protocol will determine the effectiveness of photodynamic therapy regarding the reduction of xerostomia in older adults using antihypertensive drugs.

**Trial registration:**

Clinicaltrials.gov – NCT03632096

## 1 Introduction

Systemic arterial hypertension (SAH) is a disease that affects a large percentage of the world population, including in Brazil, and is responsible for 9.4 million deaths worldwide, according to a World Health Organization (WHO) survey. Hypertension affects 30% of the Brazilian adult population, arriving at >50% in the elderly and is present in 5% of children and adolescents in Brazil, according to estimates by the Brazilian Society of Hypertension.^[1,2]^

The most frequent complications in patients with this disease are deficiencies in the function of the salivary glands, which results in a change in the quality and quantity of the saliva produced. Due to the important role that saliva plays in oral health, studies evaluating therapeutic protocols for the control and treatment of this adverse drug effect are very important.^[3]^ The low-intensity laser has been widely studied for the treatment of gland dysfunction, which can happen due to various causes.^[4,5]^

Xerostomia is a quantitative–qualitative alteration of the saliva that results in the sensation of dry mouth. This is one of the most common complaints among patients who use continuous drugs to control chronic systemic diseases, including SAH.^[6]^

This condition has been linked as side effects of several drugs that cause salivary dysfunction, including antihypertensive drugs. Diuretics are responsible for decreasing the amount of circulating intravascular and extracellular fluid in the body, including saliva, whereas anticholinergic drugs act directly on the hypothalamus, reducing salivary production.^[7,8]^ The majority of drugs used to treat this chronic disease are anticholinergics, β blockers, and diuretics, that directly influence the quantity and quality of saliva production.^[9–12]^

Antihypertensive drugs act on central alpha 2 adrenergic receptors and generally cause dry mouth. An important central area for the control of salivary secretion and the effects of alpha 2 adrenoceptor activation is the lateral hypothalamus.^[13]^ Alpha-adrenergic blockers, angiotensin-converting enzyme inhibitors, and angiotensin II receptor antagonists are classes of antihypertensives that are also associated with salivary dysfunction. Alpha-1 antagonists (tyrosine and prazosin) and alpha 2 (clonidine) antagonists are salivary flow-reducing; beta-blockers (atenolol and propranolol) also reduce salivary protein levels.^[13]^

**Figure 1:**
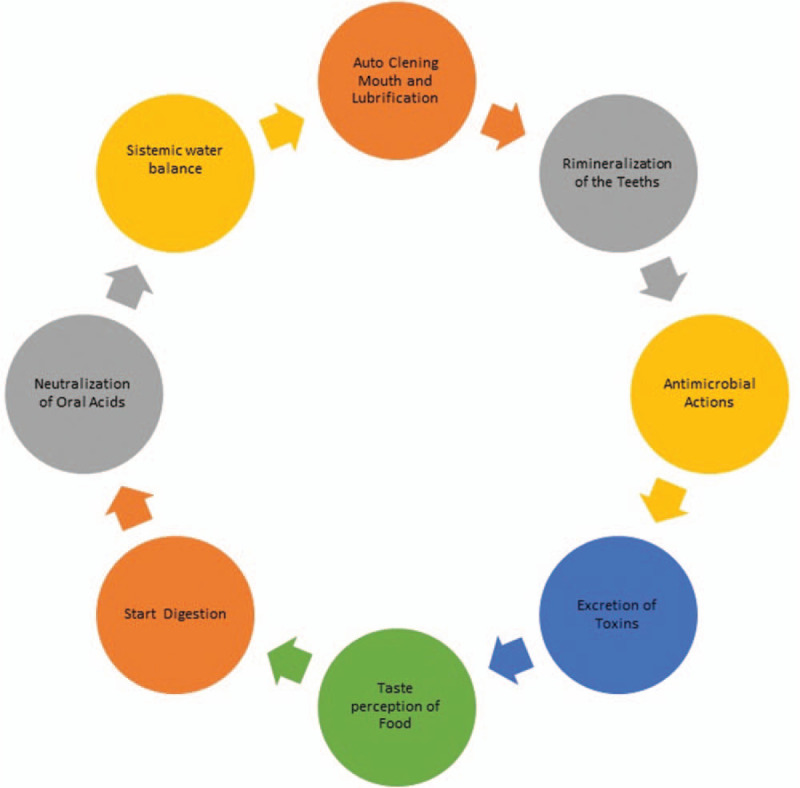
Functions of the saliva.

The secretion of salivary glands is controlled primarily by the autonomic nervous system. Parasympathetic stimulation produces abundant amounts of aqueous saliva, while sympathetic stimulation produces more viscous saliva.^[6]^Figure 1 shows the main functions of the saliva.

On the other hand, the xerostomia itself is characterized by low saliva production, but it is not necessarily related to the sensation of dry mouth. Nevertheless, it induces discomfort to the patient, interfering with phonation, chewing, starch digestion (ptyalin and salivary amylase), formation of the bolus, swallowing, dysphagia, dysgeusia, difficulty in adapting total and partial removable dentures, caries index (buffer capacity) and periodontal disease (immunoglobulin protection and self-cleaning), besides being a risk factor for bad breath and oral burning syndrome.^[6,14,15]^

In addition to the dry mouth, xerostomia can cause several conditions, such as difficulty in: swallowing food; speaking; the adaptation of partial or total prosthetics; and is also related to: burning in the mouth; halitosis, and caries.^[4,16–18]^ There are reversible causes, where there was no cellular destruction and possibility of recovery through adequate stimuli, and irreversible causes, when glandular destruction does not allow any form of reversal of the condition. In the first case, there is a decrease in salivary flow, while in the second, salivary loss is definitive.^[18]^

There are few effective methods to stimulate salivary flow, including chewing, use of systemic sialogogues, electrical stimulation, acupuncture, and the use of saliva substitutes. These methods have certain deficiencies or limitations and the most common treatment for these cases is the use of artificial saliva, frequently used and for long periods, and some types can affect the integrity of the dental enamel.

Sorbitol used in some artificial saliva formulas can trigger gastrointestinal symptoms, such as diarrhea, due to its osmotic potential. Systemic sialogogues such as pilocarpine and cevimeline hydrochloride produce pulmonary and cardiovascular side effects and may be contra indicated in certain patients according to their underlying disease.^[7,19]^

Sialometry is the most used clinical method to measure the volume of saliva, consisting of the collection of salivary fluid produced. It is considered hyposalivation when the rate of salivary flow is <1 mL/min at rest or <7 mL/min under-stimulation.^[20,21]^ The volume of secreted saliva under the patient's homeostasis conditions is approximately 1 mL/min, which may represent a volume of about 500 to 1500 mL of saliva per day. This amount and the composition of the secreted saliva can be altered in the function of systemic diseases, drugs for the treatment of chronic diseases and stress.^[21,22]^

One of the possibilities for the treatment of xerostomia is the application of photobiomodulation. Photons are absorbed by cytochromes and porphyrins in the mitochondria of the cell. A temporary release of nitric oxide from the cytochrome c oxidase binding site may occur, resulting in increased transcription and cellular respiration.^[23]^

PBM may represent a minimally invasive treatment tool for xerostomia.^[24,25]^ The likely mechanism of action of PBM is that the energy input (adenosine triphosphate [ATP]) in the acinar cells can increase the production of saliva.

The purpose of this study is present a protocol to evaluate the effect of PBM in patients with antihypertensive drug-induced xerostomia.

## 2 Methods

**Figure 2:**
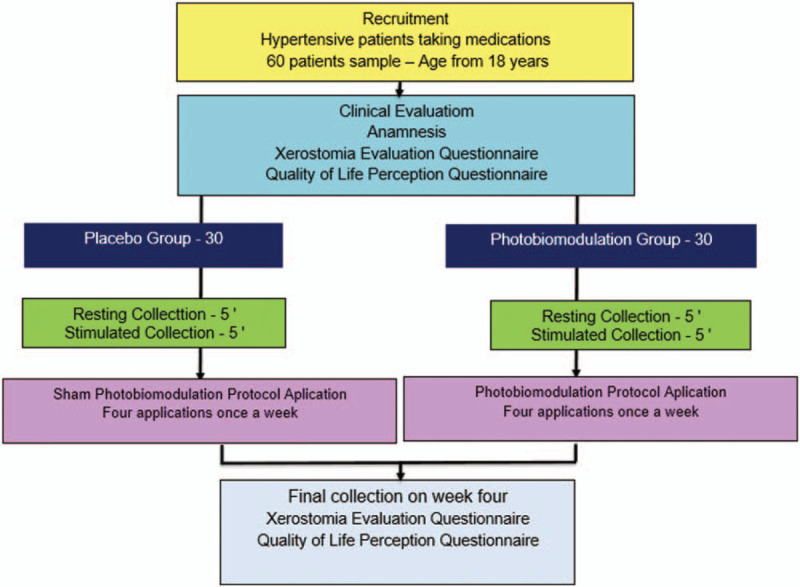
Flow of the study.

Figure 2 shows the flow of the study.

### 2.1 Study design

This is a randomized clinical trial in which all participants have the same chance of receiving treatment and will be randomly selected by using unencumbered envelopes. Participants will not know which group they belong to and the examiner should be unaware of the purpose of the study.

This research will be performed with patients who respond to the call published on the website of the University of Nove de Julho, which will offer the possibility of treatment for xerostomia in hypertensive patients who use drugs to treat the disease. It is estimated that the sample will be 60 patients. These patients will be divided into 2 groups: a placebo one and the other that will receive the PBM.

### 2.2 Trial registration

Trial registration: Clinicaltrials.gov—NCT03632096.

It was first posted and last updated in October 11, 2018.

### 2.3 Sample size

**Figure 3:**
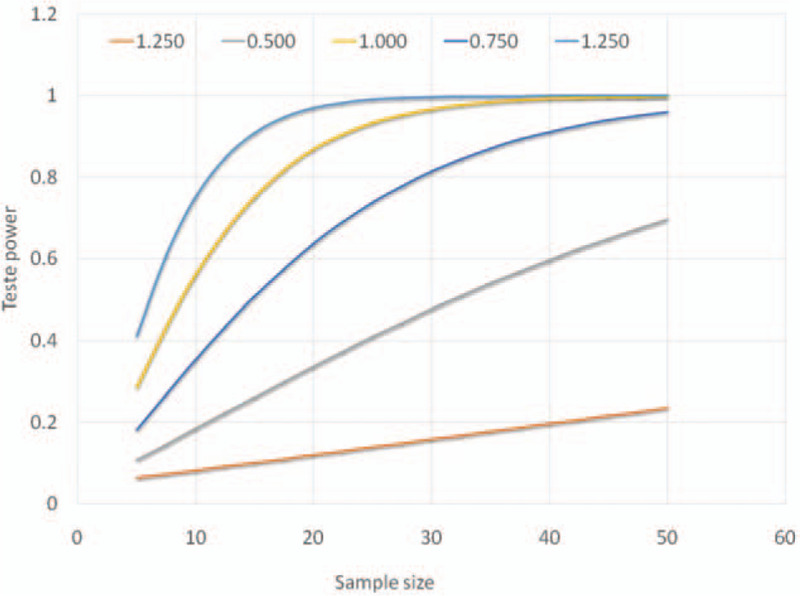
Statistical power of the hypothesis test as a function of the sample size. Each line was calculated for a different effect size.

It is known from published studies that the salivary volume (primary outcome) presents a normal distribution of probabilities, and with a variation coefficient of around 50%. Meanwhile the literature lacks information on the salivary flow difference between irradiated and non-irradiate subjects. Due to the lack of similar published works, the sample size was calculated using estimated effect size and test power as shown in Figure 3.

**Figure 4:**
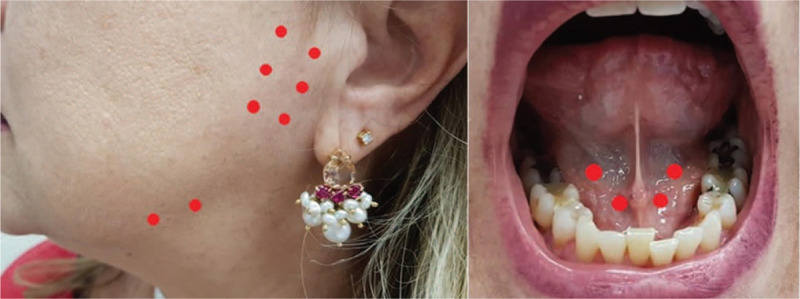
Sites of irradiation: parotid, submandibular, and sublingual salivary gland.

For the sample size analysis we assumed a 2-tailed *t* test with significant level of 0.05. Figure 4 shows that, for medium (0.750) and large (1000–1250) effect sizes, a minimum of 30 patients per group is sufficient to control statistical variance, ensuring a test power >0.80.

#### 2.3.1 Inclusion criteria

Patients over 18 years of age, hypertensive patients taking antihypertensive drugs and those who are associated with xerostomia, will be included, except for any other cause for the condition and provided they sign the free consent form. Patients with a serum level of LDL <160 mg/dL and stage 3 renal disease whose renal function reduced their total capacity by 30% to 60%, but without oral repercussion, may be part of this group.

#### 2.3.2 Exclusion criteria

Patients with cancer in the oral region, patients in radiotherapy, patients with Sjögren syndrome, diabetics, patients with stage 4 and 5 renal failure, pregnant women, infants, children under 18 years old, and those with any type of photosensitivity.

### 2.4 Recruitment and randomization

Patients who respond to the call published on the website of the University of Nove de Julho, which will offer the possibility of treatment for xerostomia in hypertensive patients who use drugs to treat the disease, will be invited to take part in the study. The 60 individuals will be randomized in 2 groups: Group A (30 individuals submitted to treatment with a PBM) and Group B (30 individuals submitted to simulated PBM). Opaque envelopes will be identified with sequential numbers (1–60) and will contain pieces of paper with the information of the corresponding experimental group (A or B). Blocked randomization will be performed in blocks of 6 patients (10 blocks for both treatments; example of a block: AABABB). All patients will have the same chance among themselves, since the method predicts randomness, noting that the participants do not know which group they belong to.

The allocation sequence will be randomized by blocks of 6 subjects in a ratio of 1:1. Each patient will receive a closed envelope to deliver to the clinician responsible for applying PBM. Patients with envelope A will undergo a placebo treatment, where all protocols of PBM application and collection of saliva will be followed, however, the laser equipment will remain off. Patients with envelope B will be submitted to the PBM protocol.

The ratings will be blind to trial participants and data analysts. As patients seek treatment service, sealed envelopes will be delivered to them. The remaining envelopes will be stored for the next patients who present for treatment.

### 2.5 Study interventions

The method consists of the application of low-intensity infrared laser in the 3 pairs of major salivary glands—parotid, submandibular, and sublingual. The parameters used will be: Laser DMC emitting at *λ* = 808 nm, 4 J/site, continuous wave, incidence of the beam perpendicular to the irradiated surface (90°) and in contact with it, resulting in irradiance of 3571 mW/site, distributed in 6 external points in each parotid, 2 external points in each sublingual, and 2 points in each submandibular (internal), totaling 20 points—16 extraoral and 4 intraoral, as shown in Figure 4.

Patients will receive 3 applications of photobiomodulation directly in the region of the 3 pairs of salivary glands already described. The detailed radiometric parameters can be observed in Table 1. The laser applications should obey the following criteria: the skin will be cleaned, the mucosa must be free of saliva, and the laser tip should come into contact with the surface to be irradiated, perpendicularly. The first application will be after the stimulating collection and the following applications will be given once a week for another 2 weeks. Two days after the end of the phototherapy will be done the final collections.

**Table 1:**
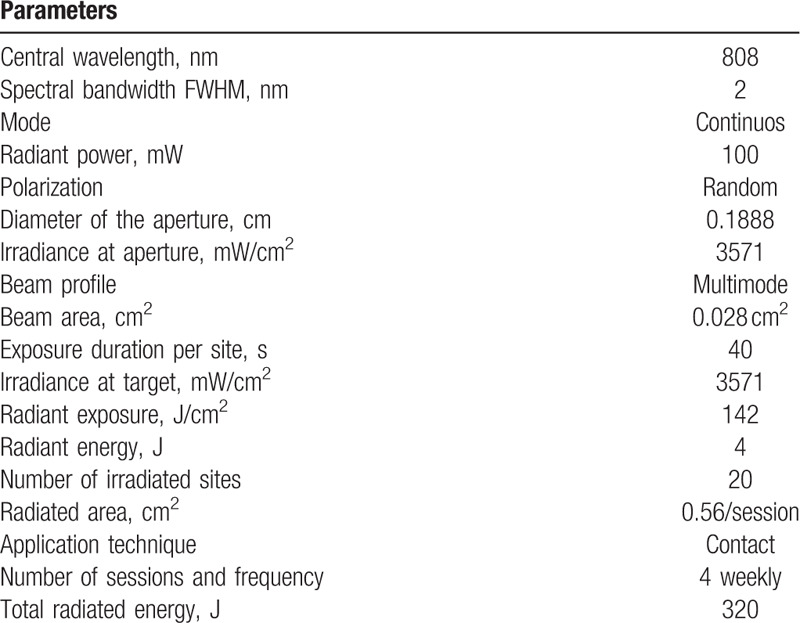
Radiometric parameters.

The placebo group will be submitted to the simulation of laser application, with the device switched off; because it is an infrared and invisible length, the patient will not be able to perceive that the equipment will be turned off. In the group that receives the PBM, the device will have its timer turned off, to avoid the perception of sound difference.

The first collection of saliva will also be done in 2 steps—unstimulated and stimulated. In the non-stimulated collection, the patient will be instructed to swallow and then lower his/her head and collect in a test tube all the saliva produced during 5 minutes. The stimulated collection will be done in the same way, however, the patient will be instructed to chew a sialogogue (silicone fragment to stimulate salivary production) for 5 minutes. After the PBM sessions (4 sessions), in the following week, the saliva will be collected again in 2 steps, as in the initial collection. The initial and final volumes will be compared to verify if there was an increase in the salivary flow. A biochemical analysis will also be made.

#### 2.5.1 Harms

No adverse effects have ever been reported while using photobiomodulation with appropriate parameters.

## 3 Study outcomes

### 3.1 Primary outcome

The primary outcome is the sialometry. The patient will be instructed to position the head forward and keep the eyes open allowing all saliva to passively pass through the collector tube, resting on the lower lip, for 5 minutes. If saliva does not drain, it will be required to be reserved in the region of the buccal floor or on the back of the tongue (without swallowing or spitting). Then, the patient will be instructed to spit out all saliva accumulated in the collection tube. The amount of saliva and foam will be evaluated and properly recorded. Then the dimethicone (removal of the air bubbles) will be used to obtain the final result. For stimulated sialometry, the patient will be asked to chew the sialogogue for 5 minutes and spit the saliva in the collection tube as it is produced.

### 3.2 Secondary outcomes

Quality of life: Patients will be submitted to anamnesis for knowledge of their general clinical picture and history of oral health, as well as intraoral clinical examination and evaluation of drugs used to control hypertension. All subjects will complete a questionnaire (OHIP 14) on quality of life and xerostomia, as well as an analysis of lip and oral mucosa dehydration.

Biochemical analysis: Analysis of salivary total proteins, urea, and calcium levels was quantified in triplicates using colorimetric analysis with commercially available kits (Bioclin, Belo Horizonte, Minas Gerais, Brazil) and a spectrophotometer (Anthos 2020—Asys - Austria). The absorbance for each marker was measured using the wavelength indicated by manufacture.

### 3.3 Statistical analyzes

The data will be analyzed regarding its distribution using the Shapiro-Wilkins test and then submitted to the appropriate tests to determine the differences between the groups. If the outcome presents data with normal distribution, the *t* test will be used. If the outcome does not present normal data, the Mann–Whitney *U* test will be used. All tests will be 2-tailed and the level of significance adopted will be *α* = 0.05.

## 4 Discussion

This study will investigate the effects of PBM in salivary glands of patients with xerostomia induced by antihypertensive drugs.

The difficulties in carrying out this study are to find patients with hypertension without other comorbidities such as diabetes, kidney disease, or Sjogrën syndrome, since about 30% of the population has several comorbidities associated with hypertension, resulting in additional difficulty in recruitment of these patients.^[19]^

Additionally, retaining the patient to the research, so that he remains the 4 weeks in treatment, without evading is a great barrier.

The target audience for this research is patients who attend the medical and dental outpatient clinics of the University of Nove de Julho. It is a target audience of low income who seeks free treatment and often the financial situation prevents the patient from returning weekly to be irradiated. It is, therefore, very important to raise awareness of this population about the risk that xerostomia poses to oral and general health, noting that these people will benefit greatly if they adhere to the project and complete the PBM cycle.

PBM can promote analgesia, cellular biomodulation, fibroblast proliferation, collagen synthesis, and tissue regeneration.^[23,26]^ Light produces a modulation effect on biological processes, with the conversion of light energy into useful energy for the cell, increasing the production of mitochondrial ATP, with increased cellular glucose consumption, intracellular calcium, and the number of mitochondria. PBM can modulate various biological processes, including inflammation, by reducing the expression of many proinflammatory cytokines.^[23,27]^

In recent years, several works have shown that ATP is a critical signaling molecule that allows cells and tissues throughout the body to communicate with each other. This new aspect of ATP as an intercellular signaling molecule broadens the understanding of the phenomenon of the universality of cytochrome c oxidase photosensitivity. Extra synthesis of ATP with monochromatic light of different wavelengths has been poorly documented for decades. This effect has long been regarded as the most useful from a phototherapy point of view.

It is known that neurons release ATP in the muscle, the gut and the bladder tissue as a messenger molecule. Receptors specific for ATP as for the signaling molecule (P2 family) and its final decomposition product, adenosine (P1 family), were found and identified. ATP activation of P2 receptors (P2X and P2Y subtypes) can produce different cellular effects. It seems that irradiation could be used as a replacement for growth factors. This field of research opens a new understanding of the complicated mechanisms of PBM. The role of ATP as a signaling molecule provides a new basis for explaining the versatility of the effects of phototherapy.^[23,26]^

A recent study with rats demonstrated that PBM increases the number of mitotic ducts of submandibular glands, suggesting the efficacy of PBM as a salivation stimulating agent in patients with xerostomia.^[28]^ PBM, using an infrared laser with radiant energy between 4 and 8 J per point, was shown to be effective in stimulating saliva production in rats and increased the concentration of secreted proteins in the parotid glands.

The literature lacks papers on the impact of PBM in patients with xerostomia induced by antihypertensive drugs. In the research carried out in the databases, no article was found that directed the PBM to patients with xerostomia induced by antihypertensive drugs. The great majority of the papers deal with xerostomia related to chemotherapy and radiotherapy.

The proposed treatments are only palliative, through artificial saliva, which presents an immediate result, but which presupposes several daily applications, with undesirable effects, according to reports of patients who use it.

## Author contributions

**Conceptualization:** Vanessa Christina Santos Pavesi.

**Formal analysis:** Sandra Kalil Bussadori.

**Funding acquisition:** Sandra Kalil Bussadori.

**Investigation:** Maria Lucia Zarvos Varellis, Vanessa Christina Santos Pavesi, Valdomiro F. Barbosa-Filho, Cícero Dayves Silva Bezerra, Felipe Gonçalves da Silva.

**Methodology:** Maria Lucia Zarvos Varellis, Anna Carolina Ratto Tempestini Horliana.

**Validation:** Daniela Fatima Teixeira Silva.

**Writing – original draft:** Maria Lucia Zarvos Varellis, Anna Carolina Ratto Tempestini Horliana, Daniela Fatima Teixeira Silva, Lara Jansiski Motta, Sandra Kalil Bussadori.

**Writing – review & editing:** Marcela Letícia Leal Gonçalves, Anna Carolina Ratto Tempestini Horliana, Daniela Fatima Teixeira Silva, Lara Jansiski Motta, Sandra Kalil Bussadori.

Alessandro Melo Deana orcid: 0000-0002-0014-6953.
